# A specific expression profile of LC3B and p62 is associated with nonresponse to neoadjuvant chemotherapy in esophageal adenocarcinomas

**DOI:** 10.1371/journal.pone.0197610

**Published:** 2018-06-13

**Authors:** Olivia Adams, Félice A. Janser, Bastian Dislich, Sabina Berezowska, Magali Humbert, Christian A. Seiler, Dino Kroell, Julia Slotta-Huspenina, Marcus Feith, Katja Ott, Mario P. Tschan, Rupert Langer

**Affiliations:** 1 Institute of Pathology, University of Bern, Bern, Switzerland; 2 Graduate School for Cellular and Biomedical Sciences, University of Bern, Bern, Switzerland; 3 Department of Visceral Surgery and Medicine, Inselspital University Hospital Bern and University of Bern, Bern, Switzerland; 4 Institute of Pathology, Technische Universität München, München, Germany; 5 Department of Surgery, Klinikum Rechts der Isar, Technische Universität München, München, Germany; 6 Department of Surgery, RoMED Klinikum, Rosenheim, Germany; University of Pittsburgh, UNITED STATES

## Abstract

Paclitaxel is a powerful chemotherapeutic drug, used for the treatment of many cancer types, including esophageal adenocarcinomas (EAC). Autophagy is a lysosome-dependent degradation process maintaining cellular homeostasis. Defective autophagy has been implicated in cancer biology and therapy resistance. We aimed to assess the impact of autophagy on chemotherapy response in EAC, with a special focus on paclitaxel. Responsiveness of EAC cell lines, OE19, FLO-1, OE33 and SK-GT-4, to paclitaxel was assessed using Alamar Blue assays. Autophagic flux upon paclitaxel treatment *in vitro* was assessed by immunoblotting of LC3B-II and quantitative assessment of WIP1 mRNA. Immunohistochemistry for the autophagy markers LC3B and p62 was applied on tumor tissue from 149 EAC patients treated with neoadjuvant chemotherapy, including pre- and post-therapeutic samples (62 matched pairs). Tumor response was assessed by histology. For comparison, previously published data on 114 primary resected EAC cases were used. EAC cell lines displayed differing responsiveness to paclitaxel treatment; however this was not associated with differential autophagy regulation. High p62 cytoplasmic expression on its own (p ≤ 0.001), or in combination with low LC3B (p = 0.034), was associated with nonresponse to chemotherapy, regardless of whether or not the regiments contained paclitaxel, but there was no independent prognostic value of LC3B or p62 expression patterns for EAC after neoadjuvant treatment. p62 and related pathways, most likely other than autophagy, play a role in chemotherapeutic response in EAC in a clinical setting. Therefore p62 could be a novel therapeutic target to overcome chemoresistance in EAC.

## Introduction

Paclitaxel (Taxol) is a member of the taxane family, which are powerful chemotherapeutic drugs, preventing cell division by disruption of microtubule function. It is widely used for the treatment of a variety of cancers, including breast, ovarian, lung and gastrointestinal carcinomas. Esophageal Adenocarcinomas (EAC) are highly malignant tumors that often are already locally and systemically advanced at the time of diagnosis [1]. Improved surgery and multimodal therapeutic concepts, including neoadjuvant chemotherapy or radiochemotherapy have improved the prognosis of EAC patients, however, the considerable high rate of resistance to conventional chemotherapy is still a major problem for the treatment of this cancer [2]. In this context, biomarkers for the prediction of response to chemotherapy could help for a proper patient selection, both for neoadjuvant and adjuvant or palliative therapy avoiding side-effects of unnecessary treatment. In addition, there is a need for therapeutic strategies aimed at overcoming chemotherapy resistance. Such approaches may encompass interference with deregulated cellular mechanisms or pathways, or targeting molecular events that are responsible for primary or acquired therapy resistance.

Macro-autophagy, in the following shortly referred to as “autophagy” is a highly regulated and conserved cellular catabolic progress that degrades and recycles cellular components such as organelles and proteins. Under basal conditions autophagy is contributing to the maintenance of cellular homeostasis. Under cellular stress, such as starvation, autophagy is a survival mechanism, as products of degradation can be recycled and reused for essential cellular processes [3]. Dysregulation of autophagy has been described in many diseases including infections, neurodegenerative diseases or heart diseases [4–6]. In cancer, autophagy has been suggested to play a dual role as it can act both pro- and anti-oncogenic. In early stages of malignant diseases, autophagy may have a tumor suppressor function because it can degrade harmful proteins and maintains genomic stability. In contrast, in later stages of malignant progression, autophagy can be used by the cancer cells as a survival mechanism that facilitates invasion, metastasis and prevents treatment induced cell death [7].

Several reports point to the potential role of autophagy for therapy resistance in EAC, mostly based on results of *in vitro* experiments. We have reported on the role of the autophagy markers LC3B and p62 in primary resected EAC in a treatment chemo-naïve setting showing that low LC3B and low p62 expression is associated with worse outcome in, chemo-naïve carcinomas.

In this study we investigated tissue samples from patients with EAC that had been treated with neoadjuvant chemotherapy before surgery, and performed additional in vitro experiments aiming at a) elucidating the role of autophagy for later therapy response b) investigate whether an induction of autophagy can be observed during treatment and c) whether different expression patterns of LC3B and p62, as well as p62 related markers Kelch-like ECH-associated protein 1 (KEAP1), nuclear factor erythroid 2 (NFE2)-related factor 2 (NRF2) and nuclear factor kappa B (NF-κB), indicating distinct forms of autophagy activation are associated with therapy response or resistance, with a particular focus on paclitaxel treatment.

## Materials and methods

### Cell lines, culture and treatment conditions

The human EAC cancer cell lines OE19, OE33, SK-GT-4 and FLO-1 from the Public Health England Culture Collections were obtained via Sigma-Aldrich, Buchs, Switzerland. OE19, OE33 and SK-GT-4 were cultured and maintained in RPMI-1640 (Sigma-Aldrich, Buchs, Switzerland, R8758) supplemented with 10% fetal bovine serum (Sigma-Aldrich, Buchs, Switzerland, F7524) and 1% penicillin streptomycin (Sigma-Aldrich, Buchs, Switzerland, P4333). FLO-1 were cultured and maintained in DMEM (Sigma-Aldrich, Buchs, Switzerland, D5796) supplemented with 10% FBS and 1% penicillin streptomycin. All cell lines were cultured in a humidified incubator containing 5% CO_2_ at 37°C.

Powdered paclitaxel (Sigma-Aldrich, Buchs, Switzerland, T7191) was reconstituted in dimethyl sulfoxide (DMSO) and stock solutions were stored at -80°C. Paclitaxel was diluted to the final concentrations indicated in the text in complete medium. Powdered Bafilomycin A1 (Enzo-Life Sciences, Lausen, Switzerland, BML-CM110) was reconstituted in DMSO and stock solutions stored at -20°C. BafA prevents autophagosome-lysosome fusion and subsequent degradation thereby inihibiting autophagy at late stages. If autophagic flux marker LC3B-II shows increased levels with the treatment of interest and BafA as compared to BafA alone this is an indication that the treatment induces autophagic flux. Autophagosomal lipidated LC3B-II migrates faster than the cytosolic unlipidated LC3B-I on an SDS-PAGE gel and therefore the two isoforms can be separated and visualized using immunoblotting. For Western blot experiments BafA was added for the last two hours of paclitaxel treatment at a final concentration of 200 nM. As a positive control for autophagy induction cells were starved by incubation with EBSS media (Sigma-Aldrich, Buchs, Switzerland, E2888) for 6hr.

### Expression plasmids, transient transfection and fluorescence microscopy

Two GFP-tagged p62 expression plasmids, GFP-p62 K7A/D69A (cytoplasmic localization) and GFP-p62 ∆303–320 K7A/D69A (nuclear localization) were kindly provided by Terje Johansen[8]. Lentiviral vectors expressing shRNA targeting p62 and a puromycin resistance gene were purchased from Sigma-Aldrich (Buchs, Switzerland, TRCN0000007234, TRCN0000007235). OE19 cells were transduced and then selected in 1.5 µg/mL puromycin as described[9]. OE19 p62 knockdown cells were plated in 6-well plates and transfected with 6 µg of p62 plasmid using Lipofectamine^TM^ 2000 (Invitrogen, Basel, Switzerland; # 11668019). Cellular localization of the two p62 proteins was assessed using GFP fluorescence. Briefly, OE19 cells were fixed with 4% paraformaldehyde and mounted in fluorescence mounting medium (SlowFadeTM Gold Antifade Mountant with DAPI, Invitrogen, Basel, Switzerland; S36938). Images were taken on an Olympus FluoView-1000 (Olympus, Volketswil, Switzerland) confocal microscope at x60 magnification.

### Alamar Blue® assay

Relative cell viability upon paclitaxel treatment was assessed using the alamarBlue® Assay (ThermoFisher Scientific, Reinach, Switzerland, DAL1100) according to the manufacturer’s instructions. The alamarBlue® reagent consists of a redox indicator, containing the dye resazurin, appearing blue in its oxidized form and red, as resazurin converted into in resorufin, in its reduced form. Metabolically active cells are cable of catalyzing this reduction resulting in colorimetric change which can be spectrophotometrically measured via absorbance. Cells were plated in 96 well flat bottom plates and allowed to adhere overnight. Cells were treated with paclitaxel and incubated with the alamarBlue for 2hr prior to the reading absorbance at 570nm and 600nm for each indicated time point. Reduction of the alamarBlue® reagent was calculated and represented as relative cell viability.

### Western blotting

Prior to lysis with urea buffer (8 M urea, 0.5% tritonX) containing protease inhibitor (complete midi, Roche Diagnostics, Rotkreuz, Switzerland) cells were washed in phosphate buffered saline (PBS). Samples were sonicated, centrifuged at 13 000 rcf for 30 minutes (min) and the resulting supernatant was transferred to a fresh tube. The Bradford protein assay (BioRad, Cressier, Switzerland) was used to determine protein concentration, 30μg of total protein per sample was denatured in selfmade 5X sample buffer with β-mercaptoethanol (Sigma Aldrich, M-7522) at 95°C for 5 min and loaded on a 4–20% stain-free pre-cast gel (BioRad). Total protein was visualized as loading control using the ChemiDoc™ MP system (BioRad). Proteins were transferred onto a polyvinylidene difluoride membrane using the Trans-Blot® Turbo™ Transfer system (BioRad) and blocked in 5% bovine serum albumin (BSA)/TBS for 1hr at room temperature (RT). The anti-LC3B antibody from Novus Biologicals (rabbit polyclonal, #NB600-1384, LuBioScience, Luzern, Switzerland) was dilution 1:1000 in 5% milk/TBS with 0.1% Tween (Sigma Aldrich, P9416) and membranes were incubated overnight at 4°C with shaking. Goat anti-rabbit horseradish peroxidase (HRP)-linked secondary antibody (Cell Signaling, Danvers MA, USA, 7076) was diluted 1:10 000 in 5% milk/TBS-T and membranes incubated for 3hr at RT with shaking, followed by 5min incubation with the ClarityTM Western ECL Substrate (BioRad, Cressier, Switzerland, 1705061) at RT with shaking. Proteins of interest were visualized using the ChemiDoc™ MP system (BioRad, Cressier, Switzerland, 1708280). Images were adjusted for brightness and quantified using ImageJ software (1.64r; NIH, Bethesda, MD, USA).

### Quantitative real-time RT-PCR (qPCR)

The miRCURY RNA Isolation Kit from Exiqon was used for RNA extraction as per the manufacturer’s instructions. RT-PCR was performed as previously described [10]. The gene expression assays Hs00215872_m1 and Hs00797944_s1 (Applied Biosystems, Rotkreuz, Switzerland) were used to quantitatively measure mRNA of WIPI1 (WD repeat domain phosphoinositide-interacting protein 1) and LC3B, respectively. HMBS was included in analysis as a housekeeping gene for normalization and primers and probes were used as previously published [11]. The ABI PRISM 7500 Sequence Detection System (Applied Biosystems, Rotkreuz, Switzerland) was used to perform measurements.

### Flow cytometry

Cell death of GFP-p62 transfected OE19 cells was assessed using flow cytometric analysis of Alexa Fluor® 647 (BioLegend, #640912) and DAPI. Data acquisition and analysis was carried out on a FACS LSR-II (BD Biosciences, Switzerland) using the FlowJo software (Ashland, OR, USA).

### Patients and tissue samples

Two different case collections of formalin fixed paraffin embedded (FFPE) archival pathology tissue were used for the immunohistochemical studies. The usage of pathological archival material for research had been approved by the local ethics commissions (Kantonale Ethikkommission Bern, Switzerland, 200/14 and Medizinische Fakultät of the Technische Universität München, 2056/08). The first case collection was generated from a total of 149 patients with locally advanced esophageal adenocarcinomas that were treated in the department of surgery of the Klinikum rechts der Isar, Technische Universität München. Neoadjuvant treatment consisted of a 5-FU and cisplatinum based chemotherapy with (n = 42) and without (n = 107) paclitaxel [12]. Esophagectomy was performed in all patients as described previously [13]. Out of this patient cohort, 127 preoperative biopsies and 83 resection specimens (including 62 matched pair samples of both biopsies and post-treatment resection specimen with residual tumor) were available for tissue analysis. For tumor categorization, the UICC TNM classification, 7^th^ edition was used[14]. Tumor differentiation (grading) was assessed on pretherapeutic, non-treated bioptic tissue. Tumor regression grading after neoadjuvant therapy was performed with standardized macroscopic and histopathologic work-up as described previously [15,16]. 12 tumors (8.1%) showed TRG1a = complete regression), 36 tumors (24.2%) TRG1b (= <10% residual tumor), 26 tumors (17.4%) TRG2 (10–50% residual tumor) and 75 tumors (50.3%) TRG3 (= >50% residual tumors). Following previous works [17] and in line with the results of the survival analysis that showed a survival benefit for patients with TRG1a, TRG1b and 2 in comparison to TRG3 (for details see below), the 74 patients with TRG1a, 1b and 2 were classified as responders, and the 75 patients with TRG3 as non-responders. Table 1 summarizes the clinic-pathological features of both the complete neoadjuvant treated cohort (n = 149) and the subcohort (n = 83), for which post-treatment resection specimens were available for analysis.

For comparison, a second case collection of primary, chemo-naïve tumors from a previous study including 114 cases was used [18]. Within this cohort, 69 cases were locally advanced tumors (i.e. pT3-4, pN any categories according to the UICC TNM classification). This allowed for a stage-related comparison between the locally advanced treatment naïve and neoadjuvant treated tumors, as well as a comparison between the primary resected cases of both case collections.

**Table 1:** Summary of patient data for complete neoadjuvant EAC treated cohort and subcohort for which post-treatment resection specimens were available for analysis.

		Total Cohort (n = 149)		Subcohort (n = 83)	
**Parameter**	**Category**	**Total**	**%**	**Total**	**%**
ypT category	ypT0	12	8.1	0	0
	ypT1	21	14.1	12	14.5
	ypT2	24	16.1	16	19.3
	ypT3	86	57.7	52	62.6
	ypT4	6	4	3	3.6
Lymph Node Metastasis	absent	57	38.3	22	26.5
	present	92	61.7	61	73.5
Distant Metastasis*	absent	123	82.6	66	79.5
	present	26	17.4	17	20.5
Grading	G1-2	65	43.6	37	44.6
	G3	84	56.4	46	55.4
Resection Status	R0	119	79.9	62	74.7
	R1	30	20.1	21	25.3
Tumor Regression Grade (TRG)	1a	12	8.1	0	0
	1b	36	24.2	15	18.1
	2	26	17.4	14	16.9
	3	75	50.3	54	65.1

* M1 category cases had supra-regional lymph node metastases which is not considered as organ metastases.

### Tissue microarray

A next generation tissue microarray (ngTMA) was constructed from tumor tissue of the 83 cases where material from the post-treatment resection specimen was available. This approach included digital annotation of scanned slides and automatic transferal of the punches was constructed from the FFPE tissue of the resection specimen with six randomly selected 0.6 mm punches from each case as described previously [19]. The immunohistochemical data from the comparison cohort were generated from a ngTMA based investigation as well [18].

### Immunohistochemical staining, scoring and subclassification

The ngTMA and the biopsies were sectioned at 4 μm. Immunohistochemical staining for LC3B and p62 was performed using an automated immunostainer (Bond RX, Leica Biosystems, Heerbrugg, Switzerland) as described previously [20]: In brief, after de-paraffination, rehydration, and antigen retrieval, the anti-LC3B antibody (Novus Biologicals #NB600-1384) was diluted 1:4000 in tris buffer and incubated at 95°C for 30 min. The anti-p62/SQSTM1 antibody (MBL rabbit polyclonal, #PM0045, LabForce, Nunningen, Switzerland) was diluted 1:9000 in tris buffer and incubated at 95°C for 30 min. The conditions for the p62 downstream targets and NF-κB were as follows: the anti-NRF2 (Santa Cruz #sc365949) was diluted 1:200 tris buffer and incubated at 95°C for 30 min, the anti-KEAP1 (Proteintech #10503-2-AP) was diluted 1:1000 tris buffer and incubated at 95°C for 30 min and the anti- NF-κB antibody (Abcam #ab7970) was diluted 1:1000 Citrate buffer and incubated at 95°C for 60 min. Visualization was performed using the Bond Polymer Refine Detection kit (Leica Biosystems, Muttenz, Switzerland, DS9800) according to the manufacturer’s instructions.

**Fig 1:**
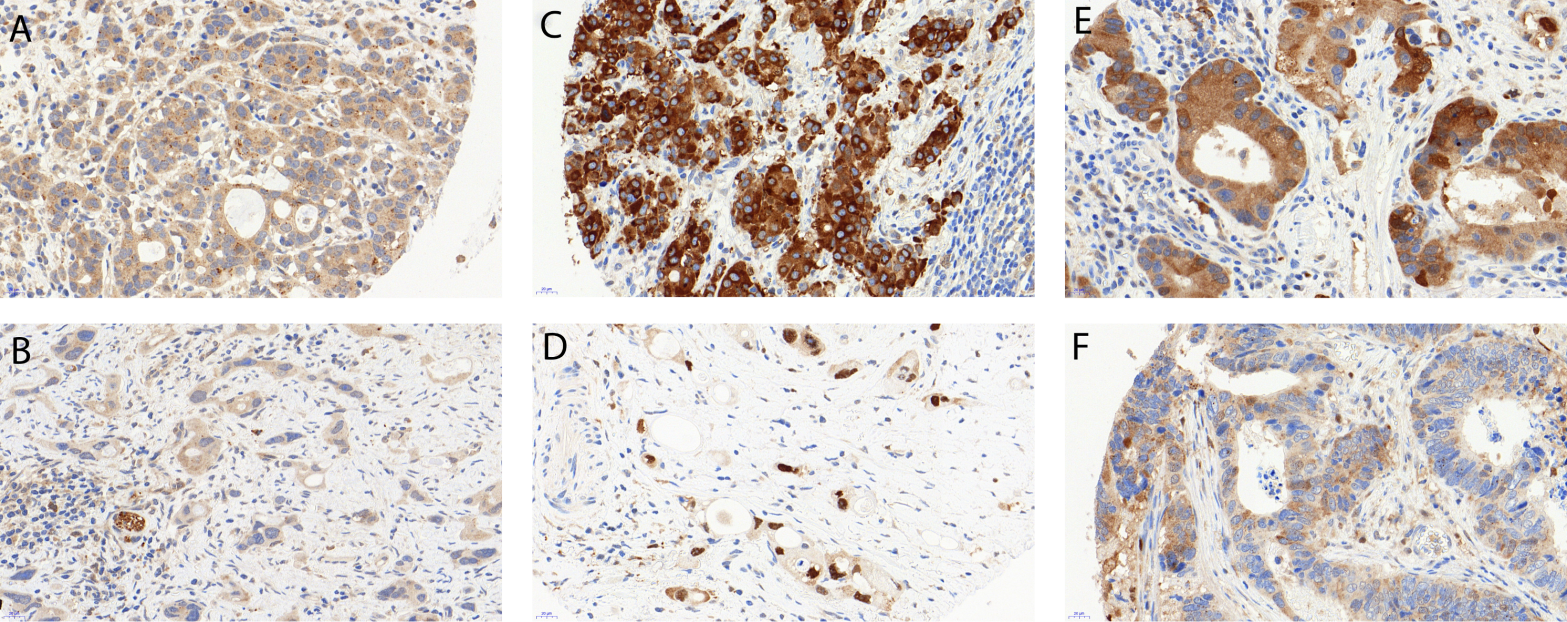
Examples of immunohistochemical stainings. (a) High LC3B dot like staining (score 2). (b) Low LC3B dot like staining (score 1), note a small nerve serving as internal positive control. (c) High p62 cytoplasmic staining (score 3), while negative nuclear staining. (d) Low p62 cytoplasmic/dot-like staining (scores 0), positive nuclear staining. (e) High cytoplasmic (score 2) and low dot-like (score 1) p62 staining. (f) Low cytoplasmic (score 1) and high dot like (score 2) p62 staining. 40x magnification for all images. Error bars indicate 20µm.

IHC staining patterns in tumors were scored across all cores by an experienced gastrointestinal pathologist (RL), as previously described, with slight modifications according to the present staining patterns [18, 20, 21]: LC3B and p62 dot-like immunohistochemical staining was scored from 0 to 3 as follows: score 0—no dots visible or barely dots visible in < 5% of the cells, score 1—detectable dots in 5–25% of the cells, score 2—detectable dots in 25–75% of the cells, score 3—dots visible in > 75% of the cells. p62, NF-κB and KEAP1 cytoplasmic immunohistochemical staining was classified as score 0—no or faint cytoplasmic staining visible, score 1—weak cytoplasmic staining visible, score 2—moderate cytoplasmic staining visible and score 3—strong cytoplasmic staining visible. p62 and NRF2 nuclear immunohistochemical staining of tumor cells was classified as score 0—nuclear staining visible in < 10% of nuclei and score 1—nuclear staining visible in > 10% of nuclei. For NRF2 there was an additional score 2 for cases with >90% strong staining of nuclei. Examples of LC3B and p62 immunohistochemical stainings are shown in Fig 1. Examples of NF-κB, NRF2 and KEAP1 stainings are shown in the S1 Fig. For further correlation with clinic-pathologic features the IHC scores were categorized as either low or high for each staining pattern as described previously, with slight modifications due to the best correlative value regarding tumor regression [18]: For LC3B and p62 dot-like and LC3B, p62, NF-κB and KEAP1 cytoplasmic staining, scores 0 and 1 were classified as low and scores 2 and 3 were classified as high. A combination score of p62 dot-like-cytoplasmic staining was calculated by adding dot-like and cytoplasmic staining. Both low expression was classified as low p62 dot-like/cytoplasmic, and mixed or both high expression was classified as high p62 dot-like/cytoplasmic staining. The dataset was also stratified into four subtypes, which have been suggested to represent different conditions of autophagy activation: low LC3B dot-like/low p62 dot-like-cytoplasmic staining (LL: basal autophagy), low LC3B dot-like/high p62 dot-like-cytoplasmic staining (LH: basal autophagy, but impaired at late stages), high LC3B dot-like/low p62 dot-like-cytoplasmic staining (HL: intact activated autophagy) and high LC3B dot-like/high p62 dot-like-cytoplasmic staining (HH activated autophagy, impaired at late stages) [18]. Nuclear staining for NRF2 was considered low for cores 0 and 1 and high for score 2.

### Assessment of T-cell infiltrates

IHC was used for the characterization and quantification of CD8 and CD3 expressing T-cell infiltration as previously described [22]. Briefly an automated immunostainer Bond III (Leica Biosystems, Germany) was used and scan slides were analyzed using the Aperio ImageScope 12.2 software (Leica Biosystems). Positive cells were counted and recorded across all TMA cores. The total cell counts of the six TMA cores was then used as the tumor infiltrating lymphocyte (TIL) count.

### Statistical analysis

For descriptive and comparative statistical analysis the SPSS 24 software (SPSS Inc, Chicago, IL, USA) was used. Associations between staining patterns and clinic-pathological parameters were evaluated using cross tabs (χ²-test or Fisher’s exact test). Binded samples were evaluated using the Wilcoxon test. Survival analysis was performed using log rank test and Cox regression analysis. The significance level was set at 0.05.

## Results

### EAC cell lines exhibit differential response to paclitaxel treatment which is not associated to differential autophagy regulation

**Fig 2:**
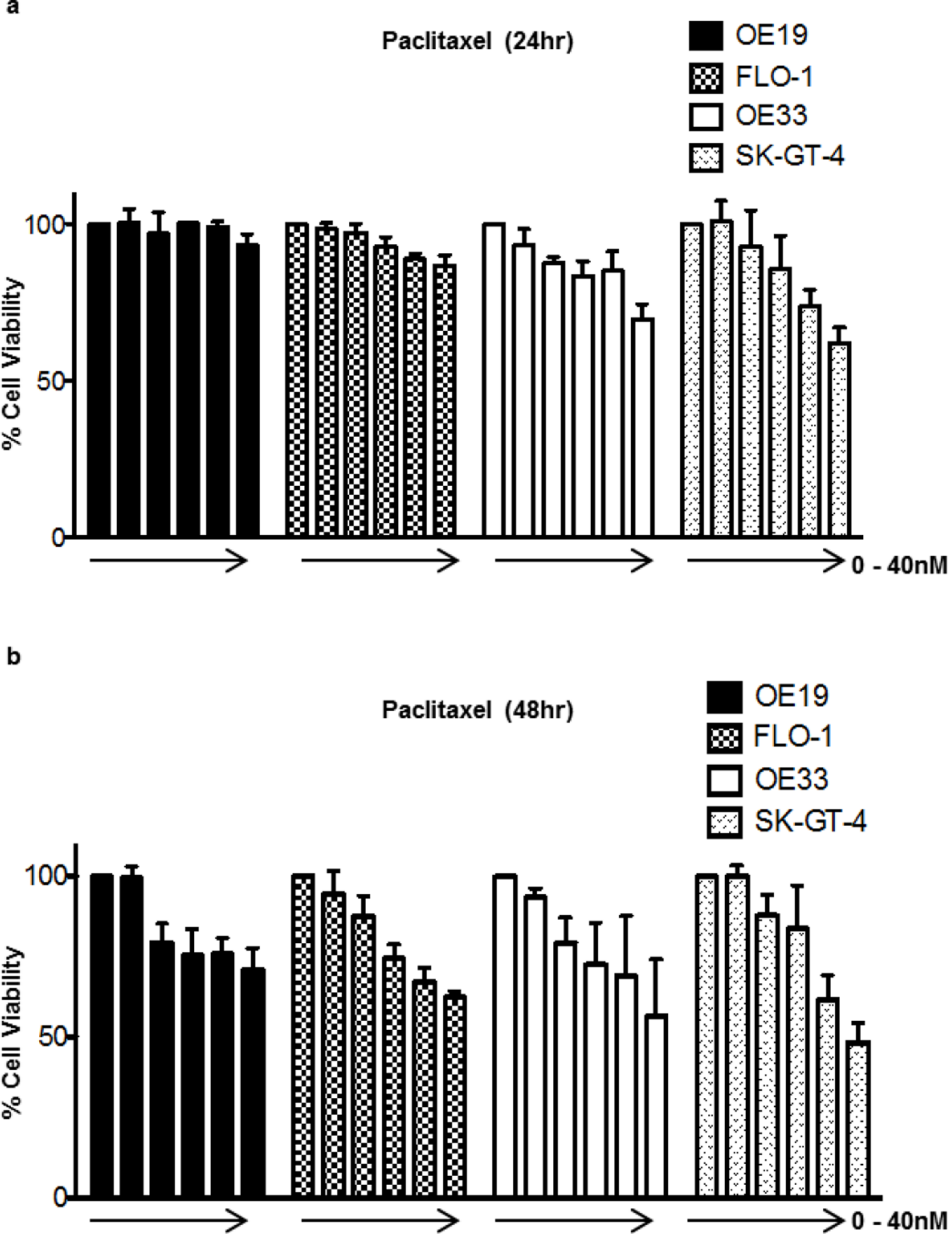
EAC cell lines exhibit differential response to paclitaxel treatment. Relative cell viability upon treatment with paclitaxel in increasing concentrations (0, 2.5, 5, 10, 20 and 40nM) was assessed using the Alamar Blue assay in OE19, FLO-1, OE33 and SK-GT-4 after 24hr (a) and 48hr (b). Error bars indicate the standard deviation of three independent experiments. The DMSO equivalent of the highest final concentration was added to the untreated condition as vehicle control and relative toxicity values were normalized to the untreated controls which were set to 100%.

**Fig 3:**
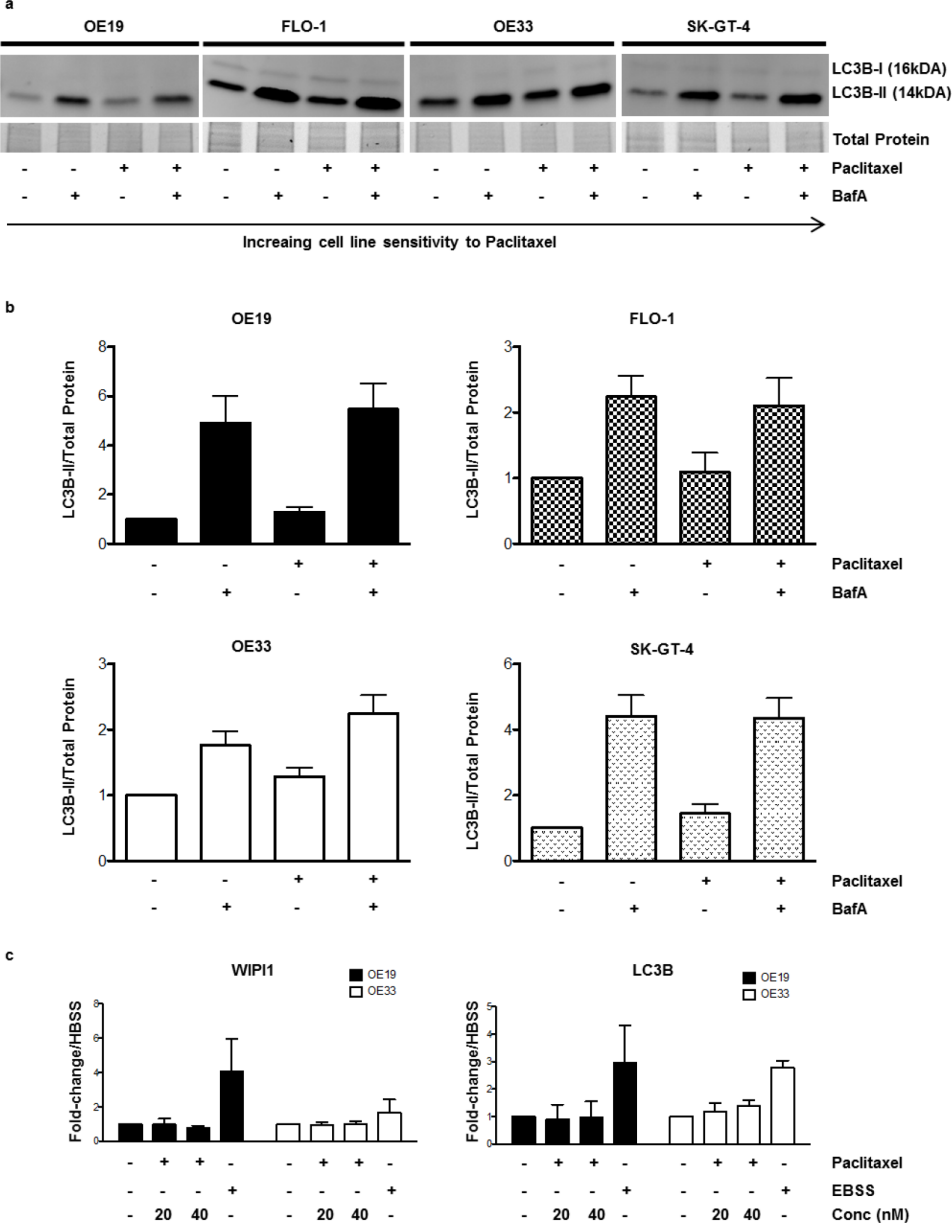
Differential response to paclitaxel is not associated with differential autophagy regulation. OE19, FLO-1, OE33 and SK-GT-4 were treated with paclitaxel, in a final concentration of 20nM, for 24hr with or without the addition of the late stage autophagy inhibitor BafA (200nM) for the last 2hr of the 24hr paclitaxel treatment. LC3B was visualized using Western blotting; total protein was used as loading control. (a) Representative blots of LC3B in all four cell lines, the LC3B-I isoform is not equally visible in all cell lines at the given exposures. (b) Quantification of the LC3B-II normalized to the total protein. Error bars indicate the standard deviation of three independent experiments. Statistical significance was not reached when conditions where compared to one another. (c) WIP1 and LC3B mRNA was assessed via qPCR upon treatment with paclitaxel at 20nM and 40nM for 24hr in OE19 and OE33. Nutrient starvation, achieved with 6hr incubation with EBSS, was included in the experimental setup as a positive control for a known autophagy inducer. Fold change was normalized to mRNA levels of housekeeping gene HBSS. The DMSO equivalent of the highest final concentration of paclitaxel was added to the untreated condition as vehicle control and relative values were normalized to the untreated controls which were set to 1. Error bars indicate the standard deviation of three independent experiments.

The relative cell viability upon paclitaxel treatment was assessed in a panel of four EAC cell lines using the Alamar Blue® assay. OE19 and FLO-1 showed the least sensitivity after 24hr, with OE33 showing a moderate response and SK-GT-4 exhibiting the highest degree of sensitivity. Similar results were observed after 48hr of treatment (Fig 2). To assess whether this observed differential drug sensitivity was associated with differential autophagy regulation or activity Western blot analysis of autophagic flux marker LC3B-II was conducted upon treatment with paclitaxel for 24hr in the absence or presence of BafA. No evidence of autophagy induction upon paclitaxel treatment was observed in OE19, FLO-1 and SK-GT-4, as LC3B-II levels upon paclitaxel treatment in the presence of BafA was equivalent to that of the BafA only condition signifying no increase of autophagic flux beyond basal levels. A slight comparative increase in LC3B-II levels were observed upon paclitaxel treatment in the presence of BafA in OE33 suggesting autophagy induction (Fig 3A and 3B). WIPI1 and LC3B mRNA were assessed via qPCR in OE19 and OE33 upon two concentrations of paclitaxel. No evidence of autophagy induction was observed as mRNA levels for both genes did not increase upon paclitaxel treatment (Fig 3C). Taken together these results indicate that the observed differential response to paclitaxel treatment is not associated with differential autophagy regulation or capacity to induce cytoprotective autophagy upon treatment.

### High cytoplasmic p62, on its own or in combination with low LC3B, is associated with nonresponse to chemotherapy with 5-FU/platinum and 5-FU/platinum + paclitaxel regimens in EAC

We previously published a report in which a cohort of primary resected treatment naïve EAC patients were assessed for the autophagy markers LC3B and p62. Both single and combination staining pattern analysis was conducted as in the present study (described in the Materials and Methods section). Of note the primary resected treatment naïve EAC cohort was subdivided into the four categories making up the *ex vivo* autophagic index also utilized in this study: low LC3B/low p62 (LL: basal autophagy), low LC3B/high p62 (LH: basal autophagy, but impaired at late stages), high LC3B/low p62 (HL: intact activated autophagy) and high LC3B/high p62 (HH: activated autophagy, impaired at late stages). One of main finding in this study was that the LL group, indicative of intact basal autophagy, faired the worse with respect to worse overall survival [18]. As a first aim we wanted to compare the autophagic prolife of the previously published primary resected EAC cohort and the current neo-adjuvant chemotherapy treated EAC cohort under investigation. Higher p62 dot-like and p62 cytoplasmic, as well as a combination of p62 dot-like and cytoplasmic expression were observed in the neo-adjuvant chemotherapy treated EAC cohort when compared to the treatment naïve primary resected EAC cohort using the same criteria for the categorization into high and low expression levels. Interestingly, the number of cases with high nuclear p62 in the treatment naïve primary resected cohort superseded that of the number of cases in the neo-adjuvant chemotherapy treated EAC cohort (Table 2). Additionally, we also assessed whether autophagic profiles between responders and non-responders significantly differed in the neo-adjuvant chemotherapy treated EAC cohort. High p62 cytoplasmic, high p62 dot-like-cytoplasmic and low/high LC3B dot-like/p62 dot-like-cytoplasmic all significantly correlated with nonresponse in the neoadjuvant chemotherapy treated EAC cohort (Table 2).

In a subsequent analysis we compared the expression patterns of the autophagy markers of entire treatment naïve EAC versus a subset of neo-adjuvant treated EAC cases treated with 5-FU/platinum + paclitaxel regimens. As with comparisons encompassing the entire neo-adjuvant treated cohort, higher p62 dot-like and p62 cytoplasmic, as well as a combination of p62 dot-like and cytoplasmic expression (p ≤ 0.001 for all), were observed in the 5-FU/platinum + paclitaxel treated subset when compared to the treatment naïve primary resected EAC cohort (Table 3).

In addition we also performed analysis comparing LC3B and p62 expression in primary resected pT3 tumors versus pretherapeutically cT3 staged that was treated with 5-FU/platinum and 5-FU/platinum + paclitaxel regimens. Again the results of this sub-analysis was in line with previous results: with p62 dot-like and cytoplasmic on its own or in combination being higher in the neo-adjuvant chemotherapy treated samples (S1 Table, p < 0.001 in all cases), the LH LC3B/p62 category was also the most represented in the neo-adjuvant chemotherapy subset (S2 Table, p < 0.001). Interesting higher LC3B dot-like staining was observed in the neo-adjuvant chemotherapy subset (S2 Table, p < 0.039).

**Table 2:** Comparison of expression of autophagy markers LC3B and p62 in primary resected and neo-adjuvant chemotherapy (nCTX) treated EAC cohorts. Significance was set to 0.05. Statistically significant p-values are shown in bold.

LC3B dots	Treatment			Total
	Primary Resected	nCTX		
		Response	Nonresponse	
**Low**	95	21	41	157
**High**	19	8	13	40
**Total**	114	29	54	197
**Treatment Naïve vs. Chemotherapy:** p-value = 0.154				
**Response vs Nonresponse:** p-value = 0.794				
**p62 dots**	**Treatment**			**Total**
	**Primary Resected**	**nCTX**		
		**Response**	**Nonresponse**	
**Low**	95	18	26	139
**High**	19	11	28	58
**Total**	114	29	54	197
**Treatment Naïve vs. Chemotherapy:** p-value < **0.001**				
**Response vs Nonresponse:** p-value = 0.256				
**p62 cytoplasmic**	**Treatment**			**Total**
	**Primary Resected**	**nCTX**		
		**Response**	**Nonresponse**	
**Low**	86 (75.4%)	21 (72.4%)	17 (31.5%)	124 (62.9%)
**High**	28 (24.6%)	8 (27.6%)	37 (68.5%)	73 (37.1%)
**Total**	114	29	54	197
**Treatment Naïve vs. Chemotherapy:** p-value < **0.001**				
**Response vs Nonresponse:** p-value < **0.001**				
**p62 nuclear**	**Treatment**			**Total**
	**Primary Resected**	**nCTX**		
		**Response**	**Nonresponse**	
**Low**	58 (50.9%)	17 (58.6%)	39 (72.2%)	114 (57.9%)
**High**	56 (49.1%)	12 (41.4%)	15 (27.8%)	83 (42.1%)
**Total**	114	29	54	197
**Treatment Naïve vs. Chemotherapy:** p-value = **0.028**				
**Response vs Nonresponse:** p-value = 0.228				
**p62 dots-cyto**	**Treatment**			**Total**
	**Primary Resected**	**nCTX**		
		**Response**	**Nonresponse**	
**Low**	76 (66.7%)	14 (48.3%)	10 (18.5%)	100 (50.8%)
**High**	38 (33.3%)	15 (51.7%)	44 (81.5%)	97 (49.2%)
**Total**	114	29	54	197
**Treatment Naïve vs. Chemotherapy:** p-value = **0.006**				
**Response vs Nonresponse:** p-value < **0.001**				
**LCB/p62**	**Treatment**			**Total**
	**Primary Resected**	**nCTX**		
		**Response**	**Nonresponse**	
**LL**	66 (57.9%)	11 (38.0%)	9 (16.7%)	86 (43.7%)
**LH**	29 (25.4%)	10 (34.5%)	32 (59.3%)	71 (36.0%)
**HL**	10 (8.8%)	3 (10.3%)	1 (1.8%)	14 (7.1%)
**HH**	9 (7.9%)	5 (17.2%)	12 (22.2%)	26 (13.2%)
**Total**	114	29	54	197
**Treatment Naïve vs. Chemotherapy:** p-value < **0.001**				
**Response vs Nonresponse:** p-value = **0.034**				

**Table 3:** Comparison of expression of autophagy markers LC3B and p62 in a primary resected EAC cohort and a subcohort of neo-adjuvant chemotherapy (nCTX) treated EAC cases with paclitaxel containing regimens. Significance was set to 0.05. Statistically significant p-values are shown in bold.

**LC3B dots**	**Treatment**		**Total**
	**Primary Resected**	**nCTX**	
**Low**	95 (83.3%)	19 (67.9%)	114 (80.3%)
**High**	19 (16.7%)	9 (32.1%)	28 (19.7%)
**Total**	114	28	142
p-value = 0.108			
**p62 dots**	**Treatment**		**Total**
	**Primary Resected**	**nCTX**	
**Low**	95 (83.3%)	9 (32.1%)	104 (73.2%)
**High**	19 (16.7%)	19 (67.9%)	38 (26.8%)
**Total**	114	28	142
p-value < **0.001**			
**p62 cytoplasmic**	**Treatment**		**Total**
	**Primary Resected**	**nCTX**	
**Low**	86 (75.4%)	12 (42.9%)	98 (69.0%)
**High**	28 (24.6%)	16 (57.1%)	44 (31.0%)
**Total**	114	28	142
p-value < **0.001**			
**p62 nuclear**	**Treatment**		**Total**
	**Primary Resected**	**nCTX**	
**Low**	58 (50.9%)	16 (57.1%)	74 (52.1%)
**High**	56 (49.1%)	12 (42.9%)	68 (47.9%)
**Total**	114	28	142
p-value = 0.674			
**p62 dots-cyto**	**Treatment**		**Total**
	**Primary Resected**	**nCTX**	
**Low**	76 (66.7%)	4 (14.3%)	80 (56.3%)
**High**	38 (33.3%)	24 (85.7%)	62 (43.7%)
**Total**	114	28	142
p-value < **0.001**			
**LCB/p62**	**Treatment**		**Total**
	**Primary Resected**	**nCTX**	
**LL**	66 (57.9%)	3 (10.7%)	69 (48.6%)
**LH**	29 (25.4%)	16 (57.1%)	45 (31.7%)
**HL**	10 (8.8%)	1 (3.6%)	11 (7.7%)
**HH**	9 (7.9%)	8 (28.6%)	17 (12.0%)
**Total**	114	28	142
p-value < **0.001**			

The analysis for the primary resected pT3 and pretherapeutically cT3 staged tumors was repeated, however this time only including tumors which have received 5-FU/platinum + paclitaxel regimens. The results were once again in line with all previous analysis (S3 Table).

### Cytoplasmic p62 renders EAC cells more resistant to paclitaxel compared to nuclear p62

**Fig 4:**
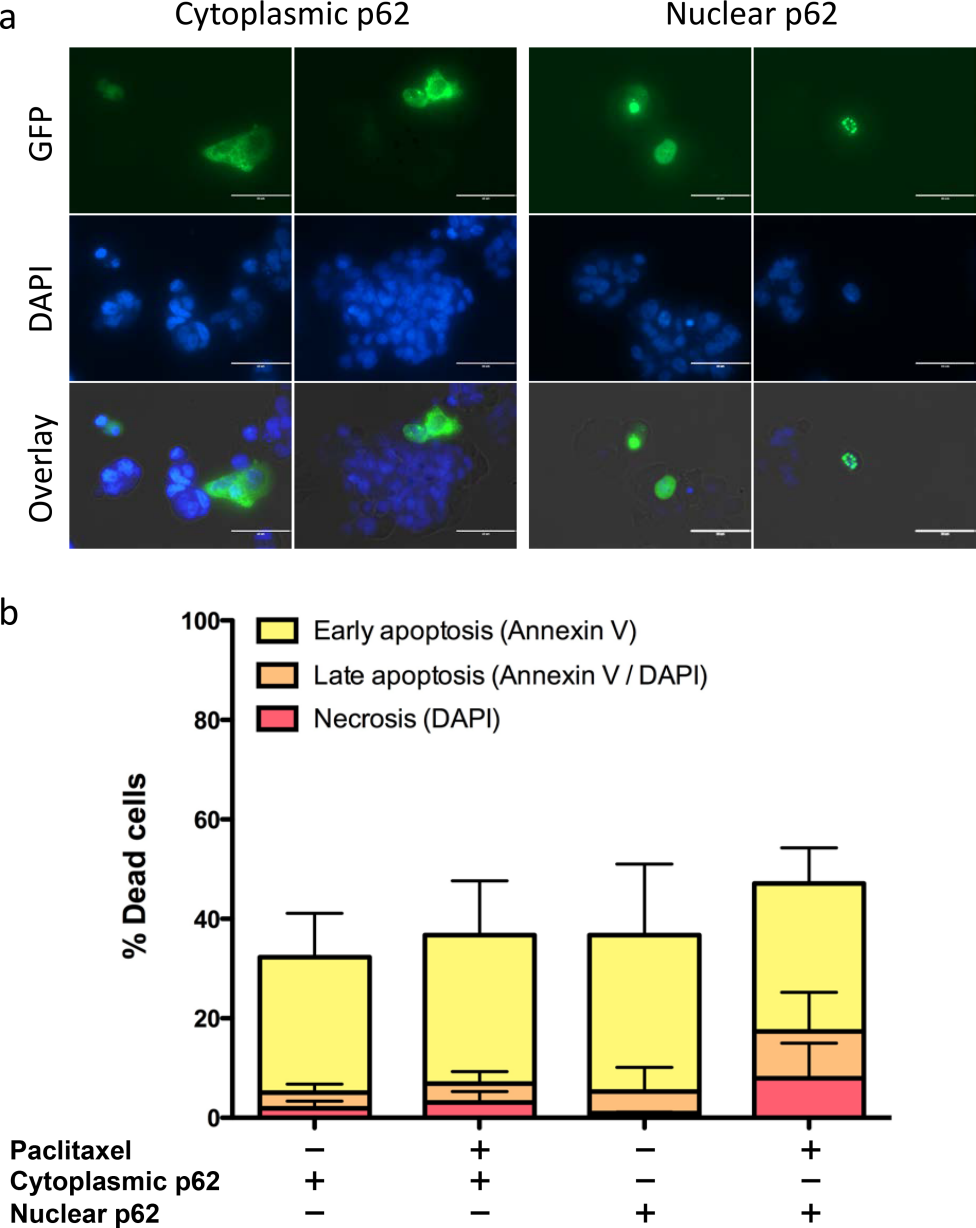
Cytoplasmic expression of p62 results in decreased responsiveness of EAC cells to paclitaxel. (a) OE19 p62 knockdown cells were transiently transfected with either a cytoplasmic or nuclear GFP-tagged p62 expression plasmid. GFP (GFP-p62 fusion proteins) and nuclear DAPI staining as analyzed by confocal microscopy are shown. (b) Annexin V/DAPI fluorescence-activated cell sorting (FACS) analysis of OE19 cells expressing cytoplasmic or nuclear p62 after 48 h of paclitaxel treatment. Bars represent four experimental replicates.

To address the question if cytoplasmic p62 contributes to paclitaxel resistance we took advantage of two mutated p62 expression plasmids allowing to assess cytoplasmic and nuclear functions of this protein. OE19 p62 knockdown cells were rescued with GFP-tagged cytoplasmic and nuclear p62 expression plasmids (Fig 4A). We found that the GFP^+^ OE19 cell fraction expressing the cytoplasmic p62 showed markedly decreased numbers of necrotic and late apoptotic cells upon paclitaxel treatment compared to OE19 cells expressing nuclear p62. Necrotic and late apoptotic OE19 cells decreased from 17.4% to 6.9% in nuclear compared to cytoplasmic p62 expressing cells (Fig 4B).

### A higher number of tumor infiltrating lymphocytes correlates with nonresponse, but does not correlate with autophagy markers in EAC *ex vivo*

Host immune response assessed by TIL counts did not correlated with the expression of either LC3B or p62 in the entire neo-adjuvant chemotherapy treated EAC cohort. It is also of note that the cell counts for CD3+ TILs in the tumor center was significantly higher in non-responders than responders (p = 0.041, data not shown). The same trend was seen for CD8+ TILs (p = 0.071, data not shown).

### No correlation between p62 expression levels and NRF2/KEAP1 or NF-κB

In order to further investigate the role of p62 for chemotherapy response, we additionally compared the expression of some downstream proteins (NRF2/KEAP1) and NF-κB in both primary resected and neoadjuvantly treated carcinomas. In both series, we could not observed a significant correlation between the expression of (nuclear) NRF2 and (cytoplasmic) KEAP1 or NF-κB with p62 expression in any of the cellular compartiments (dot-like, cytoplasmic or nuclear, data not shown).

### Tumor tissue obtained from biopsies is not suitable for response prediction or comparison with post-therapeutic samples

Assessment of pretherapeutic biopsies (n = 127) showed low pretherapeutic LC3B expression in 62 cases and 65 cases high LC3B expression. p62 dot-like staining pattern was low in 57 cases and high in 67 cases; p62 cytoplasmic staining was low in 63 cases and high in 62 cases. Nuclear positivity was observed only in 3 cases, and negativity in 122 cases (S4 Table). A positive correlation was seen for pre-and post-therapeutic LC3B expression (p = 0.26), but not for p62 dot-like, cytoplasmic or nuclear expression. There was no association between LC3B and p62 expression in the pretherapeutic biopsy tissue and histopathologic tumor response, nor survival. In order to assure reliable staining results obtained from the superficial small biopsies, we compared the staining patterns of the biopsies with those of non-treated, locally advanced EAC tissue from our previous study. The expression profiles seen in the biopsies did not match with the expected distribution across the various staining intensities, in particular with regards to the low number of nuclear positivity for p62 and the number of cases with strong cytoplasmic and dot like staining for LC3B. This discrepancy may be due to confounding factors, which may be related to local conditions such as necrosis, inflammation or ischemia or due to technical reasons such as different fixation conditions of small biopsies. Therefore, we cannot consider biopsy tissue suitable for a reliable response prediction or the comparison between pre- and post-therapeutic expression of these proteins.

### Low LC3B dot-like staining, high p62 dot-like staining and low p62 nuclear staining is associated with a worse overall survival in the neo-adjuvant treated EAC cohort

The following patho-morphologic parameters were of prognostic relevance in univariate analysis in the entire neo-adjuvant treated EAC cohort of 149 cases: ypT category (p = 0.040), presence of lymph node metastases (p<0.001), presence of distant metastases (p = 0.003), tumor differentiation (grading; p = 0.054), resection status (p<0.001) and tumor regression grade (p = 0.061; stratification into responders and non-responders p = 0.008). These parameters were also prognostic relevant in the subcohort included in the TMA analysis (n = 83): ypT category (p = 0.023), presence of lymph node metastases (p = 0.037), presence of distant metastases (p = 0.002), tumor differentiation (grading; p = 0.086), resection status (p = 0.015) and tumor regression grade (p = 0.015; stratification into responders and non-responders p = 0.005).

**Fig 5:**
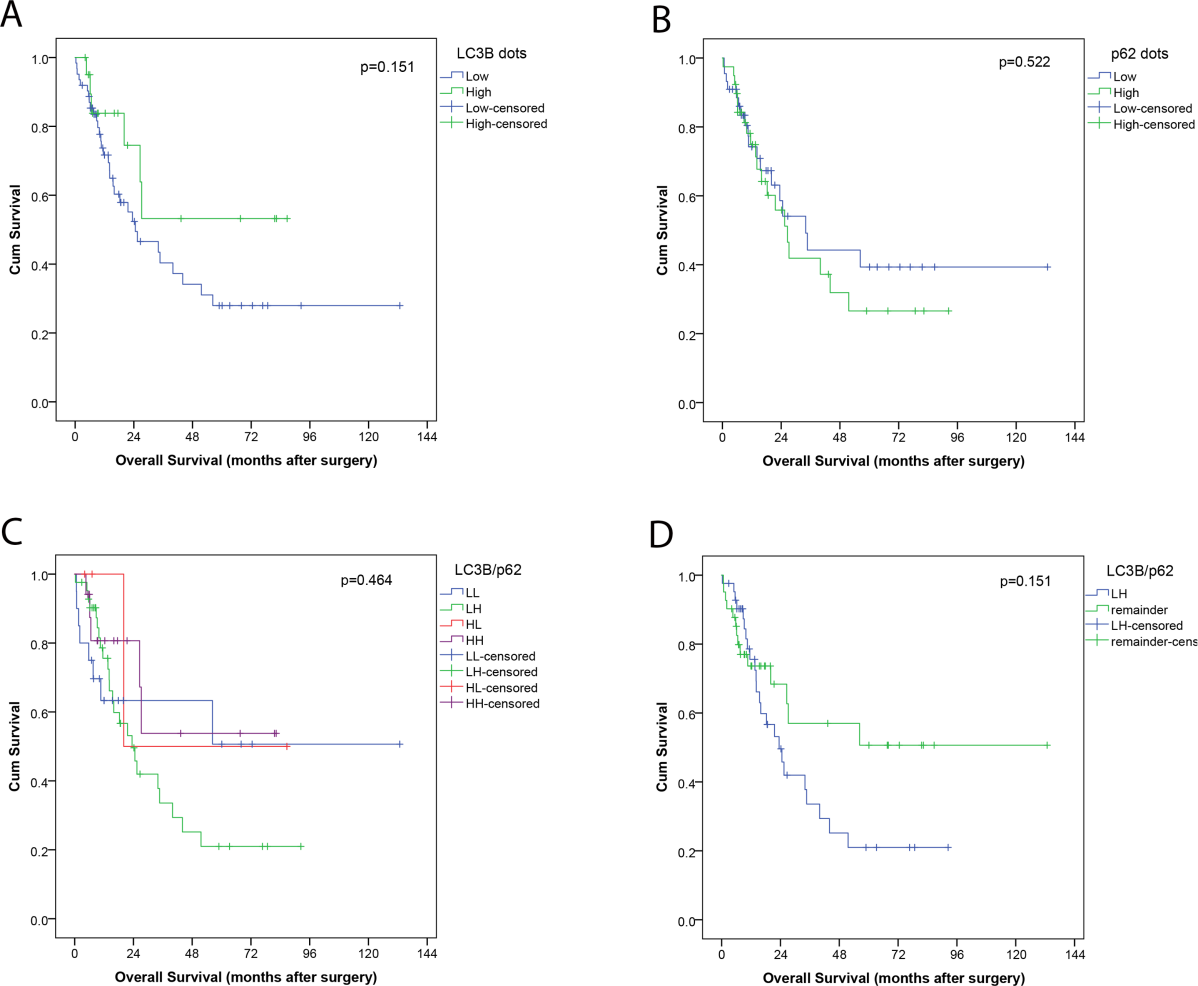
Kaplan-Meier survival curves for autophagy markers in post-treatment tumor tissue of a neo-adjuvant EAC cohort. (A) LC3B dot-like staining patterns, (B) p62 dot-like staining patterns (C) groupings of LC3B dot-like/p62 dot-like-cytoplasmic expression: Low LC3B/low p62 (LL), low LC3B/high p62 (LH), high LC3B/low p62 (HL) and high LC3B/high p62 (HH); and (D) LC3B dot-like/p62 dot-like-cytoplasmic expression LH versus remainder of all other cases. For each curve the p-value is displayed on the bottom right-hand corner.

Low LC3B dot-like staining and combined high p62 dot-like and cytoplasmic staining or low p62 nuclear staining was associated only in trend with a worse overall survival in the neo-adjuvant treated EAC cohort respectively (Fig 5, data not shown for p62 nuclear staining). It is of note that the trends observed in the univariate analysis correspond to the staining patterns that statistically correlated to nonresponse to neo-adjuvant chemotherapy. Similarly, in a univariate analysis of LC3B dot-like/p62 dot-like-cytoplasmic combination groupings showed that the LH category showed a trend of association with an unfavorable outcome when compared to other individual groups or the remaining groups collapsed into one category (p = 0.151; Fig 5), and was significantly associated with histopathological nonresponse. Moreover, low counts of CD8+ TILs, but not CD3+ were also correlated with worse overall survival (trend; p = 0.159; p = 0.65 for CD3) in univariate analysis.

### A combination of LC3B and p62 expression patterns is not a prognostic factor in a neo-adjuvant treated EAC cohort

In a multivariate analysis encompassing all factors, that showed significant associations with survival in univariate analysis, and the characterization of the neo-adjuvant EAC cohort into LH versus all other groups, the most relevant factors were presence of distant metastases p = 0.056), ypT-category (p = 0.079) and histopathological response (p = 0.119), followed by LC3B/p62 LH status (p = 0.125). Of note, in this model, no statistically significant independent prognostic factor was identified (Table 4).

**Table 4:** No statistically significant independent prognostic factor was identified in a neo-adjuvant chemotherapy treated EAC cohort. HR–Hazard Ratio.

parameter	HR	95% confidence interval		p-value
		min	max	
ypT category	1.722	0.939	3.157	0.079
Lymph node metastases absent vs present	0.964	0.604	1.538	0.876
distant metastases absent vs present	2.362	0.977	5.706	0.056
Grading G1-G2 vs G3	1.288	0.510	3.252	0.592
Resection Status (R) R0 vs R1	0.944	0.385	2.316	0.901
Histopathological Response TRG1-2 vs TRG3	2.004	0.836	4.801	0.119
LC3B/p62 LH vs LL/HL/HH	0.485	0.193	1.221	0.125

## Discussion

In the present study we investigated the role of autophagy in neo-adjuvant treatment response in EAC, with a particular focus on paclitaxel, *in vitro* and *ex vivo*. We observed differential responsiveness to paclitaxel treatment in a panel of four EAC cell lines. However, we found no evidence that the observed differential response to paclitaxel *in vitro* is associated with differential autophagy regulation. Accumulation of the LC3B-II upon paclitaxel in the presence of late-stage autophagy inhibitor BafA when compared to paclitaxel alone, which is indicative of autophagy induction, was not observed. When comparing the levels of the autophagy markers in the treatment naïve versus the neo-adjuvant treated cohort significantly higher levels of p62 dot-like and cytoplasmic, on their own and in combination, were observed in the neo-adjuvant retreated cases. Assessing the autophagy markers LC3B and p62 in a treatment naïve EAC and neo-adjuvant treated EAC cohort, high p62 cytoplasmic on its own or in combination with p62 dot-like expression correlated with nonresponse. Additionally, the combination of low LC3B dot-like/high p62 dot-like-cytoplasmic expression correlated with nonresponse.

O’Donovan *et al*. reported that cytoprotective autophagy was induced in an EAC cell line resistant to 5FU and cisplatinum [23]. This can be interpreted as being in contrast to our observations as differential response to paclitaxel treatment in EAC cell lines did not correlate to differential autophagy regulation. However, Maskey *et al*. reported that while etoposide and cisplatinum, DNA damaging agents, induced autophagy in the Jurkat T lymphocyte cell line, paclitaxel and nocodazole, drugs which exact their anti-neoplastic via acting on microtubules, did not induce autophagy [24]. Despite the differing cell systems, this would be in line with our current data and would suggest that the induction of autophagy, whether cytoprotective or not, seems to be dependent on the mode of action of the cytotoxic drug. It is of note to mention that autophagy as a survival and resistance mechanism against paclitaxel treatment has been described in many other tumor entities, highlighting the fact that the role of autophagy in chemotherapeutic response is tumor type specific [25–27].

Other publications investigating the role of autophagy in EAC are scarce. A recent follow up study demonstrated that the pharmacological autophagy inducers rapamycin and lithium show diverging effects when combined with chemotherapeutics agents in esophageal cancer cell lines [28]. Lower levels of the early autophagy initiator Beclin 1 was reported in dysplastic Barrett’s esophagus and EAC, when compared to non-dysplastic Barrett’s esophagus and non-neoplastic mucosa [29]. LC3B ring-like and LC3B stone-like structures were reported in another neoadjuvant treated EAC cohort which had prognostic significance [30]. In contrast to this study, but in line with our previous work, and most probably due to different antibodies used in these studies we did not observe any ring-like or stone-like LC3B staining patterns in our neoadjuvant treated EAC cohort.

Previously we published that low p62 expression correlates with a more aggressive phenotype, worse prognosis and worse overall survival in the treatment naïve primary resected EAC cohort also featured in this study [18]. It is therefore of particular interest that high p62 in the cytoplasmic compartment on its own or in combination with low LC3B dot-like expression correlates with nonresponse and is significantly higher in the neo-adjuvant chemotherapy treated cohort compared to the treatment naïve cohort. Importantly, our cell line experiments support these clinical findings since cytoplasmic expression of p62 in an EAC cell line resulted in decreased paclitaxel sensitivity compared to EAC cells expressing nuclear p62. As low LC3B/High p62 dot-like-cytoplasmic expression can be indicative of basal autophagy impaired at late stages, it can be interpreted that in nonresponding tumors basal autophagy is blocked upon neo-adjuvant chemotherapy. Given that autophagy has also been described to contribute to apoptosis it can be hypothesized that basal autophagy competence is a prerequisite for therapy induced apoptotic cell death, hence impairment correlating to nonresponse. The non-autophagic functions of p62 could also be contributing factors to our clinical observations.

p62 is a key player in NRF2-KEAP1-antioxidant response element (ARE) pathway and p62 upregulation in this context has been described as contributing to oncogenesis [31,32]. As increased NRF2 signaling can potentially result in an increase of the expression of pro-survival ARE genes, and a reduction of reactive oxygen species (ROS). This would also be a mechanistic explanation for high p62 expression correlating to nonresponse as p62 facilitates the autophagy degradation of KEAP1, the negative regulator of NRF2. p62 can also act as pro-inflammatory player in the NF-κB pathway. Pro-inflammatory conditions are also considered to be tumorigenetic and could contribute to nonresponse as opposed to therapy induced cell death. We additionally investigated the association between LC3B and p62 in the post-treatment tumor tissues and found no significant correlations. Moreover, we did not find any significant correlation between p62 expression in any of the cellular compartments and expression levels of KEAP1, NRF2 and NF-κB. We therefore could not establish a potential mechanistic link between the clinical observations of p62 levels and its functions in these pathways. The observation that the number of cases with high nuclear p62 in the primary resected cohort superseded that of the number of cases in the neo-adjuvant chemotherapy treated cohort could potentially speak to nuclear p62’s role as a mediator of an alternative nuclear proteolytic degradation pathway[8].

In addition to LC3B, p62 is ubiquitously used as a marker of autophagic flux in cancer research. However few studies focus on the mechanistic role of p62, nor the predicative and prognostic power of p62. Some studies show that high expression of p62 in gastrointenstinal cancers in a treatment naïve setting is associated with a more aggressive or advanced phenotype as well as with a worse overall survival [33,34]. This can be seen as in contrast to previous work done in a treatment naïve setting, where low p62 was associated with a worse prognosis [18]. Few studies focusing on p62 in a neo-adjuvant chemotherapeutic setting in gastrointestinal and other cancers have been published. Park *et al*. found that p62 was overexpressed in the majority of tumors in their neo-adjuvant 5FU chemotherapy treated colon carcinoma cohort. However p62 expression did not correlate to any clinic-pathological features nor was it prognostic in univariate and multivariate analysis [35]. The study by Huang *et al*. demonstrated that autophagy induction, specifically accompanied by down regulation of p62, contributed to decreased chemotherapy induced death in colon cancer cell lines [36]. However, this study was done in the presence of therapeutic inhibition of mechanistic target of rapamycin (mTOR), the chief negative regular of autophagy, thus in a different context as our current study. Yu and colleagues reported that upregulation of p62 contributed to cisplatinum resistance in ovarian cancer *in vitro* via clearance of ubiquitinated proteins [37]. If we consider p62 staining in the cytoplasmic compartment alone, this would be in line with our current findings that higher levels of p62 correlate with nonresponse. However if we consider the groupings with LC3B, this would be rather in contrast as the group indicative of basal autophagy impaired at late stages correlated with nonresponse as well.

The recent genomic characterization of esophageal cancer by The Cancer Genome Atlas (TCGA) Research Network revealed EAC is most genetically similar to the chromosomally instable (CIN) subtype of gastric carcinoma (GC) [38]. Therefore we also looked to the body of work done in GC with respect to the role of autophagy and chemotherapeutic response. Most of the recent publications, using *in vitro* cell line based and *in vivo* mouse based models, are in line with the work in EAC reporting autophagy as a survival and resistance mechanism to DNA damaging agents [39]. For example, the upregulation of ATG5 was reported to be associated with chemoresistance [40]. Moreover, multiple publications demonstrate that inhibiting autophagy potentiates the cytotoxic effects of DNA damaging chemotherapeutic drugs [41–44]. Interestingly, Yang and colleagues observed contradictory phenomena as they published that 5FU possibly suppresses a microRNA which negatively regulates autophagy via Beclin1, which results in increased autophagic flux and autophagic cell death [45]. Studies investigating autophagy in GC patient tissue in a neo-adjuvant chemotherapeutic setting are sparse [39]. It is also of importance to mention that the aforementioned studies all preceded the TCGA publication and molecular subtyping of GC was not taken into consideration in most cases, making correlations and comparisons with EAC studies difficult.

The fact that we had to exclude the biopsies as a viable option to assess autophagy markers before therapy is a limitation of this study. The comparison of matched pre- and post-therapeutic samples would have been most informative; however, we did perform a comparative analysis with a different treatment naïve primary resected cohort. The subset of cases treated with 5-FU/platinum + paclitaxel regimens is rather small in number, however the results of all our sub-analyses was all in line with those performed with the entire neo-adjuvant treated collective. This suggests that this is not a paclitaxel related phenomena but a general trend regardless of composition of chemotherapy regimens. Using IHC in patient tissue to assess such a dynamic process as autophagy can also be considered as a limitation as this only represents a snapshot in the disease state. However, our strategy of using combination of both LC3B and p62 to create an autophagic index is a more biologically informative strategy.

In summary, we describe in this study that higher p62 expression correlates with nonresponse to neo-adjuvant chemotherapy in EAC. Furthermore, our observation that an autophagic profile which can be interpreted as a basal autophagy impaired at late stages also corresponds to nonresponse is a novel finding. As with previously published data in a treatment naïve setting [18], the autophagy independent roles of p62 in chemotherapeutic response cannot be ignored and warrants further investigation. There is also a good basis of evidence which points to the fact that the mode of action of cytotoxic drugs may be important in autophagic regulation, which should be taken into consideration with respect to future therapeutic strategies involving modulation of autophagy in combination with conventional chemotherapy. We observed no differential autophagy regulation upon differing responses to paclitaxel in EAC *in vitro*. Moreover, results of the sub-analysis done on the subset of EAC cases receiving 5-FU/platinum + paclitaxel regimens were in line with the results from the entire neo-adjuvant chemotherapy. Therefore in our cohort differential expression patterns correlating to response may be a phenomenon related to DNA damaging agents (5-FU/platinum) and not paclitaxel. The underlying mechanisms of our current observations requires elucidation to advance future autophagy or p62 targeted therapy modulation.

## Supporting information

S1 Fig Examples of immunohistochemical stainings. (a) KEAP-1 high expression (score 2), (b) KEAP-1 low expression (score 0), (c) NFKB high expression (score 3) (d) NFKB low expression (score 1) E NRF-2 high expression (score 2) F NRF-2 low expression (score 1). Error bars indicate 50µm.
(TIF)

S1 Table Frequency of individual IHC scores for LC3B dot-like, p62 dot-like, p62 cytoplasmic and p62 nuclear staining in a neo-adjuvant chemotherapy treated EAC cohort. (DOCX)

S2 Table Comparison of expression of autophagy markers LC3B and p62 in treatment naïve primary resected EAC and neo-adjuvant chemotherapy treated EAC T3 tumors. Significance was set to 0.05. Statistically significant p-values are shown in bold.
(DOCX)

S3 Table Comparison of expression of autophagy markers LC3B and p62 in treatment naïve primary resected EAC and subset of neo-adjuvant paclitaxel containing chemotherapy treated EAC neo-adjuvant chemotherapy treated EAC T3 tumors. Significance was set to 0.05. Statistically significant p-values are shown in bold.
(DOCX)

S4 Table Frequency of individual IHC scores for LC3B dot-like, p62 dot-like, p62 cytoplasmic and p62 nuclear staining in pre-therapeutic biopsies of a neo-adjuvant chemotherapy treated EAC cohort. (DOCX)
