# Correction: A specific expression profile of LC3B and p62 is associated with nonresponse to neoadjuvant chemotherapy in esophageal adenocarcinomas

**DOI:** 10.1371/journal.pone.0224832

**Published:** 2019-10-30

**Authors:** Olivia Adams, Félice A. Janser, Bastian Dislich, Sabina Berezowska, Magali Humbert, Christian A. Seiler, Dino Kröll, Julia Slotta-Huspenina, Marcus Feith, Katja Ott, Mario P. Tschan, Rupert Langer

The seventh author’s name is spelled incorrectly. The correct name is: Dino Kröll.
