# Supplementary material for: A specific expression profile of LC3B and p62 is associated with nonresponse to neoadjuvant chemotherapy in esophageal adenocarcinomas
Source: PLoS One. 2018 Jun 13;13(6):e0197610. doi: 10.1371/journal.pone.0197610 (PMC5999293; doi:10.1371/journal.pone.0197610)
Supplement: S1 Table — (DOCX) [file pone.0197610.s002.docx]

**S1 Table**

| **Staining** | **Individual Scores** | | | | **Total** |
| --- | --- | --- | --- | --- | --- |
|  | **0** | **1** | **2** | **3** |  |
| **LC3B dots** | 28 | 34 | 21 | 0 | 83 |
| **p62 dots** | 8 | 36 | 37 | 2 | 83 |
| **p62 cyto** | 2 | 36 | 40 | 5 | 83 |
| **p62 nuclear** | 56 | 27 | N/A | N/A | 83 |

N/A – not applicable
