# Supplementary material for: A specific expression profile of LC3B and p62 is associated with nonresponse to neoadjuvant chemotherapy in esophageal adenocarcinomas
Source: PLoS One. 2018 Jun 13;13(6):e0197610. doi: 10.1371/journal.pone.0197610 (PMC5999293; doi:10.1371/journal.pone.0197610)
Supplement: S3 Table — Significance was set to 0.05. Statistically significant p-values are shown in bold. (DOCX) [file pone.0197610.s004.docx]

**S3 Table**

| **LC3B dots** | **Treatment** | | **Total** |
| --- | --- | --- | --- |
|  | **Treatment Naïve**  **Primary Resected** | **Neo-adjuvant Chemotherapy** |  |
| **Low** | 61 | 19 | 80 |
| **High** | 8 | 9 | 17 |
| **Total** | 69 | 28 | 97 |
| p-value = **0.036** | | | |
| **p62 dots** | **Treatment** | | **Total** |
|  | **Treatment Naïve**  **Primary Resected** | **Neo-adjuvant Chemotherapy** |  |
| **Low** | 59 | 9 | 68 |
| **High** | 10 | 19 | 29 |
| **Total** | 69 | 28 | 97 |
| p-value < **0.001** | | | |
| **p62 cytoplasmic** | **Treatment** | | **Total** |
|  | **Treatment Naïve**  **Primary Resected** | **Neo-adjuvant Chemotherapy** |  |
| **Low** | 52 | 12 | 64 |
| **High** | 17 | 16 | 33 |
| **Total** | 69 | 28 | 97 |
| p-value = **0.004** | | | |
| **p62 nuclear** | **Treatment** | | **Total** |
|  | **Treatment Naïve**  **Primary Resected** | **Neo-adjuvant Chemotherapy** |  |
| **Low** | 40 | 16 | 56 |
| **High** | 29 | 12 | 41 |
| **Total** | 69 | 28 | 97 |
| p-value = 1.000 | | | |
| **p62 dots-cyto** | **Treatment** | | **Total** |
|  | **Treatment Naïve**  **Primary Resected** | **Neo-adjuvant Chemotherapy** |  |
| **Low** | 48 | 4 | 52 |
| **High** | 21 | 24 | 45 |
| **Total** | 69 | 28 | 97 |
| p-value < **0.001** | | | |
| **LCB/p62** | **Treatment** | | **Total** |
|  | **Treatment Naïve**  **Primary Resected** | **Neo-adjuvant Chemotherapy** |  |
| **LL** | 43 | 3 | 46 |
| **LH** | 18 | 16 | 34 |
| **HL** | 5 | 1 | 6 |
| **HH** | 3 | 8 | 11 |
| **Total** | 69 | 28 | 97 |
| p-value < **0.001** | | | |
