# Supplementary material for: A specific expression profile of LC3B and p62 is associated with nonresponse to neoadjuvant chemotherapy in esophageal adenocarcinomas
Source: PLoS One. 2018 Jun 13;13(6):e0197610. doi: 10.1371/journal.pone.0197610 (PMC5999293; doi:10.1371/journal.pone.0197610)
Supplement: S4 Table — (DOCX) [file pone.0197610.s005.docx]

**S4 Table**

| **Staining** | **Individual Scores** | | | | **Total** |
| --- | --- | --- | --- | --- | --- |
|  | **0** | **1** | **2** | **3** |  |
| **LC3B dots** | 15 | 47 | 57 | 8 | 127 |
| **p62 dots** | 3 | 54 | 66 | 1 | 124 |
| **p62 cyto** | 6 | 57 | 62 | 0 | 125 |
| **p62 nuclear** | 122 | 3 | N/A | N/A | 125 |

N/A – not applicable
